# *Helicobacter pylori* infection in pregnant women in four districts of Uganda: role of geographic location, education and water sources

**DOI:** 10.1186/1471-2458-14-915

**Published:** 2014-09-04

**Authors:** Rhona Kezabu Baingana, John Kiboko Enyaru, Lena Davidsson

**Affiliations:** Department of Biochemistry and Sports Science, College of Natural Sciences, Makerere University, P. O. Box 7062, Kampala, Uganda; Kuwait Institute for Scientific Research, Food and Nutrition Program, Environment and Life Sciences Research Center, Kuwait, Saudi Arabia

**Keywords:** *Helicobacter pylori*, Uganda, Pregnant women, Geographical, Ethnicity, Water, Education, Rural, Urban

## Abstract

**Background:**

The prevalence of *Helicobacter pylori* infection varies in relation to geography, ethnicity and socioeconomic factors. Available data on the prevalence of *Helicobacter pylori* infection in Uganda are not representative of the general population. We sought to describe the epidemiology of this infection in pregnant women in Uganda to provide background data for a study into the effect of *H. pylori* infection during pregnancy on the hematological response to iron supplementation.

**Methods:**

Using a cross-sectional design, *H. pylori* infection was assessed by the stool antigen test among 447 pregnant women attending antenatal care clinics in Apac, Mbale, Mbarara and Rakai Districts which are in different geographical regions in Uganda, and at Kawempe Health Center which serves a low-income densely populated area in Kampala City. Socio-demographic and household data were collected by face-to-face interviews using a questionnaire. Associations between *H. pylori* infection and socio-demographic and household characteristics were analyzed using logistic regression.

**Results:**

The overall prevalence of *H. pylori* infection was 45.2% but varied by geographical location from 18.2% in Apac District to 60.5% at Kawempe Health Centre. At 18.4%, the Langi ethnic group, who were enrolled exclusively in Apac District, had the lowest prevalence of *H. pylori* infection while the Gisu had the highest prevalence (58.4%). *H. pylori* was independently associated with enrollment at clinics not in Apac (adjusted OR = 5.68; 95% CI: 3.02-10.7) and with using water from public wells, boreholes or springs (AOR = 3.20; 95% CI: 1.19-8.61) and from rivers, lakes or streams (AOR = 5.20; 95% CI: 1.58-17.05). Urban residence (AOR = 1.71; 95% CI: 1.13-2.60) and no formal education (AOR = 1.95; 95% CI: 1.03-3.67) were also independently associated with *H. pylori* infection.

**Conclusions:**

The unexpected variation in the prevalence of *H. pylori* infection in Uganda calls for population-based studies in the region and offers an opportunity to study the transmission dynamics of *H. pylori* infection. The association between *H. pylori* infection and surface water sources for household use suggests waterborne transmission of *H. pylori* infection highlighting the need for concerted efforts in environmental health in communities and at the household level.

## Background

*Helicobacter pylori* infection has a major role in the development of gastritis and peptic ulcers and is an important risk factor for gastric cancer [1]. The prevalence of *H. pylori* infection is persistently higher in developing countries than in developed countries [2] and can vary by ethnicity [3–5], place of birth, and socioeconomic factors even among persons living in the same country [6]. Data on *H. pylori* infection in Uganda are limited and are not representative of the general population: the prevalence was 74% in patients with dyspepsia referred for endoscopy [7] and 86% in patients with cancer and benign tumors [8]. Additional data are from a low-income urban setting in Kampala City where the prevalence of *H. pylori* infection in children twelve years and below was 44.3% based on a stool antigen test [9] and was 63% based on a serological test in children aged 1–10 years [10]. While these data are informative, the coverage in terms of geographical location and age-group is limited. Additionally, to our knowledge, there is no data from apparently healthy, non-referred adults.

There is compelling evidence from case studies, observational studies and interventional trials in children and non-pregnant adolescents and adults suggesting a role for *H. pylori* infection in the etiology of anemia [11, 12]. However, the evidence for a similar role in anemia in pregnancy remains inconclusive [13–17]. Because available data on *H. pylori* infection in Uganda are limited and are not representative of the general population, we set out to study the prevalence of *H. pylori* infection in pregnant women in Uganda so as to provide background data for a study into the effect of *H. pylori* infection on the hematological response to iron supplementation in pregnant women in Uganda.

Among the tests available for the diagnosis of *H. pylori* infection, the invasive ones (endoscopy with biopsy for histology, culture, and rapid urease test) are not suitable for pregnant women. Serological tests do not discriminate between current and past infections. The noninvasive “gold standard” ^13^C urea breath test was not available for this study for logistic reasons. The stool antigen test offers a simple, yet robust alternative [18, 19] which has been recommended by the European *Helicobacter* Study Group as one of two non-invasive tests (the other being the urea breath test) [20]. We used the stool antigen test to determine *H. pylori* infection and described the association of *H. pylori* infection with well-known risk factors including rural–urban residence, sources of water for household use and educational attainment in pregnant women attending antenatal care at health facilities in various regions in Uganda.

## Methods

### Study setting and design

**Figure 1:**
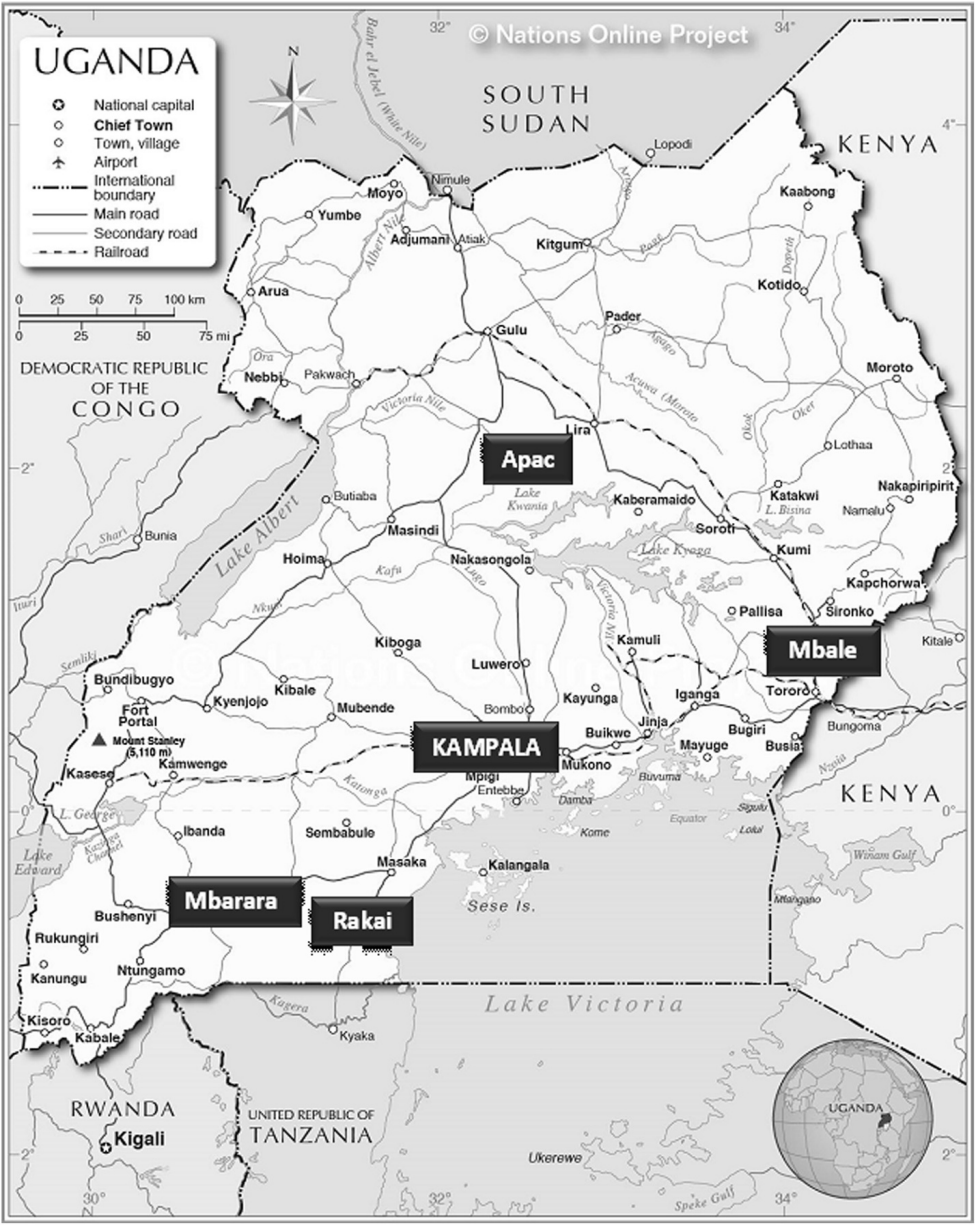
**Map of Uganda showing study areas.**

Uganda, a land-locked country in East Africa has over 40 geographically localised ethnic groups. The Baganda in the south-central region form the largest group, comprising almost 18% of the population. The other major ethnic groups are Banyankole (10%), Basoga (9%,) Iteso (8%), Bakiga 7%, Langi (6%), Bagisu (5%), Acholi (5%) [21]. This was a cross-sectional study in which four districts: Apac, Mbale, Mbarara and Rakai, were purposively selected from the pilot districts of the *Innovations at Makerere Committee* program to represent different geographical locations and the major ethnic groups in the country (Figure 1). Kawempe Health Centre in Kampala District was purposively selected for comparison as a health facility that serves a densely-populated, low-income area. Within each district, four health facilities were purposively selected to represent rural and urban settings and to include a wide geographic area within the district.

### Study population and sample size

The study population consisted of pregnant women attending antenatal care clinics (ANC) at health facilities in Apac (Apac Hospital, Alenga Health Centre (HC) III, Abongomola HC III Aboke HC III), Mbale (Mbale Hospital, Bududa Hospital, Bunambale HC II Busano HC II), Mbarara (Bwizibwera HC IV, Bugamba HC IV, Kinoni HC IV, Mbarara MC HC IV) and Rakai (Rakai Hospital, Lyantonde Hospital, Kibale HC II, Bethlehem HC II) and at Kawempe HC IV in Kampala District. According to the 2006 and 2011 Uganda Demographic and Health Surveys 94% of pregnant women attend antenatal care at least once [22, 23], thus pregnant women attending ANC are representative of pregnant women in our setting.

Available data at the time of planning the study indicated that the prevalence of *H. pylori* infection was 74% in patients with dyspepsia referred for endoscopy in Uganda [7] and ranged from 70% to 100% in adults in Africa [24, 25]. A sample size of 322 women would be needed to estimate a prevalence of 70% with a confidence level of 95% and a precision of 5% [26]. However, because this was the first study in apparently healthy, non-referred adults in Uganda, and because we wanted to achieve a wider coverage of the country compared to a previous study [7], our aim was to recruit about 500 women.

### Data collection

Data were collected from Rakai and Mbale Districts from August to October 2005, from Kawempe Health Centre in February and March 2006 and from Mbarara and Apac Districts from February to April 2008. The protracted data collection period was due to logistics challenges.

On each day of data collection, the background, aims and procedures of the study were first explained to the health workers of the antenatal care clinic and then to the women attending the clinic collectively. The women who came for antenatal care on the days of data collection at each health facility were approached consecutively to participate in the study. Any woman who had pregnancy-related complications or reported that she used antibiotics in the two weeks preceding data collection was excluded from the study. Informed written consent was given by each subject, and the study was performed according to the Declaration of Helsinki. The study was approved by Makerere University Faculty of Medicine Research and Ethics Committee and research clearance was obtained from Uganda National Council for Science and Technology.

After receiving consent, socioeconomic and demographic data were collected through interviews using a questionnaire. Information collected included the women’s age, parity, reported gestational age, educational level and employment status. We also assessed household characteristics such as household size, number of rooms, sources of water for household use, housing materials and ownership of household assets. Consenting women were given clearly labeled stool sample bottles for self-collection of stool. Women who were unable to provide the stool sample on the spot were requested to bring it at the next scheduled data collection visit if it was feasible for them to return. Stool samples were transported to the Department of Biochemistry at Makerere University in Kampala, Uganda on ice-packs in a cool box and were analyzed the same day or were stored in a refrigerator (4-8°C) or frozen (-20°C) pending analysis. None of the samples was kept for more than 48 hours at 4-8°C or for more than 7 days at -20°C before analysis. Stool samples were analyzed using a *H. pylori* stool antigen test (Premier Platinum HpSA, Meridian Diagnostics Inc., Cincinnati, OH, USA) following the manufacturer’s instructions. According to the manufacturer, the test is 96.1% sensitive and 95.7% specific. In brief, diluted stool samples were put into microplate wells followed by peroxidase-conjugated antibody. The wells were incubated for 1 hour at room temperature then washed to remove any unbound material, and a substrate was added to each well. After 10 minutes incubation, stop solution was added and the result determined spectrophotometrically at 450/630 nm as per the manufacturer’s recommendations. Positive and negative controls were run with each assay. Results were expressed as *H. pylori* positive or negative.

### Data analysis

Principal components analysis was used to assign a weight to the following household assets: type of house (mud and wattle or cement and bricks), nature of floor (packed soil or cement/tiles), type of roof (grass-thatched or iron sheets/tiles); ownership of a watch, radio, bicycle, mobile phone, land, domestic animals, a motorised vehicle (car or motorcycle) and television, subject’s employment status and whether the household hired laborers. The Kaiser-Meyer-Olkin Measure of Sampling Adequacy which reflects the proportion of common variance in the variables was 0.79 while Barlett’s Test of Sphericity was statistically significant, indicating significant relationships between the variables. Four components with eigenvalues higher than 1 were extracted; the first accounted for 27.8% of the total variance and was selected as the socio-economic score. The socio-economic score was ranked and categorized into wealth quintiles (Lowest, Second, Middle, Fourth, Highest).

Cross-tabulation was used to obtain frequencies and proportions of background characteristics by location and of *H. pylori* infection by background characteristics. Univariate logistic regression was used to assess crude associations between *H. pylori* infection and risk factors. Given that ethnic groups in Uganda are geographically localized, there is a high correlation between ethnic group and district in our data (*r* = 0.358, *p* = 0.000). Thus, ethnic group was not included in the subsequent analysis. Bivariate logistic regression was used to assess the relationship of *H. pylori* infection to risk factors whilst controlling for location. The risk factors associated with *H. pylori* following univariate and/or bivariate analysis (at *p* ≤ 0.05) were entered into a multivariable logistic regression to select the final set of independent risk factors. The final multiple logistic regression model fit was determined by the Hosmer-Lemeshow test statistic [27]. A model fits the data if the Hosmer-Lemeshow statistic has a *p* > 0.05. All statistical analyses were performed using SPSS 13.0 for Windows (SPSS Inc).

## Results

Of the 502 women who consented, 38 did not provide stool samples. Seventeen stool samples were omitted for poor quality, leaving a total of 447 women in the study. The demographic and household characteristics are summarized in Table 1. Just over half of the women were rural residents and 61% were 24 years of age or less. Only 12.5% of the women did not have any formal education and 94.6% used pit latrines. About two-thirds collected water for household use from public wells, boreholes or springs. Apac had the highest proportion of women in the lowest wealth quintile (58%) while Kawempe Health Centre had the highest proportion of women in the highest wealth quintile (69%).

**Table 1:** **Demographic and household characteristics by location**

Characteristic		Kawempe		Mbale		Mbarara		Rakai		Apac		Total	
		(N = 86)		(N = 107)		(N = 105)		(N = 61)		(N = 88)		(N = 447)	
		n	%	n	%	n	%	n	%	n	%	n	%
Residence	Urban	86	100	65	60.7	10	9.1	50	82.0	35	39.8	246	55.0
	Rural	0	0	42	39.3	95	90.9	11	18.0	53	60.2	201	45.0
Age (years)	15-19	18	20.9	25	23.4	25	23.6	10	16.7	17	19.3	95	21.3
	20-24	41	47.7	36	33.6	42	39.6	25	41.7	34	38.6	178	39.9
	25-29	18	20.9	24	22.4	15	14.2	11	18.3	16	18.2	84	18.8
	30-34	5	5.8	16	15.0	16	15.1	10	16.7	12	13.6	59	13.2
	35-39	4	4.7	5	4.7	6	5.7	4	6.7	5	5.7	24	5.4
	40-44	0	0	1	0.9	2	1.9	0	0	3	3.4	6	1.3
Married		72	83.9	100	93.5	99	94.5	56	91.8	79	98.5	406	90.8
Education	None	5	5.7	16	15.0	11	10.1	8	13.1	16	18.2	56	12.5
	Primary+	81	94.3	91	85.0	94	89.9	53	86.9	72	81.8	391	87.5
Parity	≤ 2	67	77.9	55	51.4	68	65.1	36	59.0	44	50.0	270	60.4
	3+	19	22.1	52	48.6	37	34.9	25	41.0	44	50.0	177	39.6
Toilet	None	1		5	4.7	0	0	0	0	3	3.4	9	2.0
	Flush	6	6.9	6	5.6	2	1.9	1	1.6	0	0	15	3.4
	Pit latrine	79	92.0	96	89.7	103	98.1	60	98.4	85	96.6	423	94.6
Household crowding	Low	3	3.5	12	11.2	19	18.2	9	14.8	6	6.8	49	11.0
	Medium	50	58.1	79	73.8	80	76.4	42	68.9	58	65.9	309	69.1
	High	33	38.4	16	15.0	6	5.5	10	16.4	24	27.3	89	19.9
Ethnic group	Ganda	55	64.0	2	1.9	0	0	36	59.0	0	0	93	20.8
	Lango	0	0	0	0	0	0	0	0	87	98.9	87	19.5
	Gisu	3	3.5	98	91.6	0	0	0	0	0	0	101	22.6
	Nkole	18	20.9	0	0	103	98.2	20	32.8	0	0	141	31.5
	Others	10	11.6	7	6.5	2	1.8	5	8.2	1	1.1	25	5.6
Wealth Index	Lowest	0	0	15	14.0	18	17.3	10	16.4	51	58.0	94	21.0
	Second	0	0	26	24.3	24	22.7	7	11.5	26	29.5	83	18.6
	Middle	2	2.3	43	40.2	22	20.9	15	24.6	7	8.0	89	19.9
	Fourth	25	28.7	14	13.1	31	29.1	15	24.6	3	3.4	88	19.7
	Highest	59	69.0	9	8.4	11	10.0	14	23.0	0	0	93	20.8
Water source	Home tap	5	5.7	5	4.7	2	1.8	9	14.8	2	2.3	23	5.1
	Public tap	44	51.7	17	15.9	33	31.2	7	11.5	1	1.1	102	22.8
	Public WBS	35	40.2	78	72.9	66	63.3	35	57.4	66	75.0	280	62.6
	RLS	2	2.3	7	6.	4	3.7	10	16.4	19	21.6	42	9.4

WBS = well, borehole or spring; RLS = River, lake or stream.

### *H. pylori*infection and demographic and household characteristics

*Helicobacter pylori* antigens were found in the stool of 202 of the 447 women who provided stool samples, giving a prevalence of 45.2% (95% Confidence Interval (CI) 40.3-50.1%). The prevalence ranged from 18.2% (95% CI: 10.2-26.3%) in Apac to 60.5% (95% CI: 50.2-70.8%) at Kawempe Health Centre. Geographic location, ethnic group, residence and parity were associated with *H. pylori* infection (Table 2). At 18.4%, the prevalence of *H. pylori* infection among the women enrolled in Apac was significantly lower than the prevalence in the other locations (Table 2) thus we used Apac as the reference category when controlling for location. Women enrolled at Kawempe Health Centre had 7-fold higher odds of *H. pylori* infection compared to women enrolled in Apac (Table 2), while the odds of infection were 3 to 5-fold higher in women enrolled in the other districts compared to women enrolled in Apac. With the exception of Kampala, which is the capital city and therefore has the highest ethnic diversity, there is a high correlation between ethnic groups and location in Uganda. Accordingly, the odds of *H. pylori* infection in the ethnic groups followed a similar pattern to the odds of infection based on location. Women enrolled at urban health facilities had 1.79 times higher odds of *H. pylori* infection compared to women enrolled at rural centers; this difference reduced but remained significant after controlling for location.

**Table 2:** **Prevalence of** ***H. pylori*** **infection by demographic and socio-economic variables**

Variable (No. of women)		***H. pylori***+	Univariate	***p***	Adjusted for location	***p***
		% (95% CI)	OR (95% CI)		AOR (95% CI)	
Location	Apac (88) (Ref)	18.2 (10.2-26.3)	1.00		-	-
	Kawempe Health Centre (86)	60.5 (50.2-70.8)	7.02 (3.51-14.01)	0.00	-	-
	Mbale (107)	55.1 (45.7-64.5)	5.53 (2.85-10.73)	0.00	-	-
	Mbarara (105)	42.9 (33.4-52.4)	3.45 (1.78-6.70)	0.00	-	-
	Rakai (61)	49.2 (36.7-61.7)	4.36 (2.08-9.11)	0.00	-	-
	All without Apac (359)	51.8 (46.6-57.0)	4.89 (2.74-8.73)	0.00	-	-
Ethnic group	Lango^1^ (87) (Ref)	18.4 (10.3-26.5)	1.00		-	-
	Nkole^2^ (142)	46.5 (38.3-54.7)	3.85 (2.04-7.27)	0.00	-	-
	Gisu^3^ (101)	58.4 (48.8-68.0)	6.23 (3.19-12.20)	0.00	-	-
	Ganda^4^ (93)	55.9 (45.8-66.0)	5.63 (2.85-11.10)	0.00	-	-
	Others^5^ (24)	37.5 (18.1-56.9)	2.96 (1.13-7.78)	0.03	-	-
Residence	Rural (202) (Ref)	37.3 (30.9-44.3)	1.00		1.00	
	Urban (246)	51.6 (45.4-57.8)	1.79 (1.23-2.62)	0.00	1.58 (1.06-2.34)	0.03
Age (years)^6^	15-19 (95) (Ref)	53.2 (43.1-63.3)	1.00		1.00	
	20-24 (178)	44.9 (37.6-52.2)	0.72 (0.44-1.19)	0.20	0.71 (0.42-1.20)	0.20
	25-29 (84)	42.2 (31.6-52.8)	0.64 (0.35-1.16)	0.14	0.63 (0.34-1.17)	0.14
	30-34 (59)	37.3 (25.0-49.6)	0.52 (0.27-1.02)	0.06	0.51 (0.26-1.03)	0.06
	35-39 (24)	45.8 (28.3-64.2)	0.76 (0.30-1.83)	0.52	0.76 (0.30-1.94)	0.56
	40-44 (6)	16.7 (0.9-58.1)	0.18 (0.02-1.57)	0.12	0.24 (0.03-2.35)	0.22
Marital status	Not married^7^ (39)	46.2 (30.6-61.8)	1.00		1.00	
	Married (406)	45.3 (34.4-68.6)	0.97 (0.50-1.88)	0.93	0.91 (0.46-1.82)	0.79
Education status	Primary and above (392) (Ref)	43.9 (39.0-48.8)	1.00		1.00	
	None (56)	55.4 (42.4-68.4)	1.59 (0.90-2.79)	0.11	1.98 (1.08-3.63)	0.03
Parity	≤ 2 (270)	49.3 (43.3-55.3)	1.00		1.00	
	3+ (176)	38.6 (31.4-45.8)	0.64 (0.44-0.95)	0.03	0.71 (0.47-1.05)	0.09
HCI^8^	Low (50)	52.0 (38.2-65.8)	1.00		1.00	
	Medium (309)	44.7 (34.3-45.3)	0.75 (0.41-1.36)	0.34	0.81 (0.44-1.49)	0.50
	High (89)	43.8 (41.9-62.7)	0.72 (0.36-1.44)	0.35	0.88 (0.43-1.81)	0.73
Water source	Own tap (23) (Ref)	40.9 (20.8-61.0)	1.00		1.00	
	Public tap (104)	52.5 (42.9-62.1)	2.94 (1.08-8.06)	0.04	2.71 (0.98-7.49)	0.05
	Public well, borehole or spring (280)	43.3 (37.5-49.1)	2.25 (0.86-5.88)	0.10	2.90 (1.09-7.68)	0.03
	River, lake, or stream (41)	48.8 (33.5-64.1)	2.70 (0.87-8.22)	0.08	5.45 (1.67-17.80)	0.01
Toilet	None (8) (Ref)	37.5 (4.0-71.0)	1.00		1.00	
	Flush (15)	33.3 (9.4-57.2)	0.63 (0.11-3.41)	0.59	0.39 (0.06-2.26)	0.29
	Pit latrine (423)	45.9 (41.2-50.6)	1.06 (0.28-4.00)	0.93	0.86 (0.21-3.56)	0.84
Wealth Quintile	Lowest (94) (Ref)	34.0 (25.2-44.1)	1.00		1.00	
	Second (83)	44.6 (34.4-55.3)	1.57 (0.86-2.86)	0.15	0.85 (0.43-1.69)	0.65
	Middle (89)	43.8 (34.0-54.2)	1.54 (0.84-2.85)	0.16	0.76 (0.38-1.54)	0.45
	Third (88)	46.6 (36.5-56.9)	1.74 (0.94-3.19)	0.08	0.74 (0.37-1.52)	0.42
	Highest (93)	54.8 (44.7-64.6)	2.44 (1.33-4.48)	0.00	1.00 (0.49-2.04)	0.99

^1^Lango and Acholi; ^2^Nkole, Kiga, Nyarwanda, Bafumbira; ^3^Gisu, Banyole, Luuya, Bagwere; ^4^Ganda, Baruuli, Soga, Bakooki; ^5^Tooro, Alur, Nubbi, Iteso, Lugbara, Tanzanian, Nyoro, Bakonjo, Karamajong, Japadhola. ^6^Mean age = 24.1 years, range 15–42 years. ^7^Not married includes never married, widowed and divorced.

^8^HCI = Household Crowding index: ratio of the number of persons in the household to the number of rooms in the house. Ref = Reference category.

The odds of *H. pylori* infection among women with no education relative to women with some formal education was not significant in the univariate analysis, however, when location was controlled for, the odds ratio was 1.98 (95% CI: 1.08-3.63, *p* = 0.03) relative to women with some formal education. Women from the highest wealth quintile had higher odds of *H. pylori* infection relative to women from the lowest quintile (OR = 2.44; 95% CI: 1.33-4.48, *p* = 0.00); controlling for location eliminated this relationship. After controlling for location, women who get water from public wells, boreholes or springs and from rivers, lakes or streams respectively had 3 and 5-fold higher odds of infection relative to women who have their own taps at the household. The other household characteristics were not associated with *H. pylori* infection.

### Multivariable model

Urban residence (adjusted OR = 1.71; 95% CI: 1.13-2.60, p = 0.02) and no formal education (AOR = 1.95; 95% CI: 1.03-3.67, p = 0.04) each had an independent and positive association with *H. pylori* infection (Table 3). Enrollment outside Apac (AOR = 5.68; 95% CI: 3.02-10.7, p = 0.00) and using water from public wells, boreholes or springs (AOR = 3.20; 95% CI: 1.19-8.61, p = 0.02) and from rivers, lakes or streams (AOR = 5.20; 95% CI: 1.58-17.05, p = 0.01) were also independently positively associated with *H. pylori* infection.

**Table 3:** **Logistic regression model of** ***Helicobacter pylori*** **infection on predictor variables**

Characteristic	Adjusted ^1^OR (95% CI)	***p***
Location		
Apac (Ref)	1.00	
All without Apac	5.68 (3.02-10.7)	0.000
Residence		
Rural (Ref)	1.00	
Urban	1.71 (1.13-2.60)	0.017
Education status		
Primary and above (Ref)	1.00	
None	1.95 (1.03-3.67)	0.040
Water source		
Own tap	1.00	
Public tap	2.74 (0.99-7.61)	0.053
Public well, borehole, spring	3.20 (1.19-8.61)	0.022
River, lake, spring (Ref)	5.20 (1.58-17.05)	0.007

^1^Adjusting for location, residence, education, water source.

## Discussion

In this sample of pregnant women attending ANC clinics at selected health facilities in Uganda in the districts of Apac, Mbale, Mbarara and Rakai and at Kawempe Health Centre in Kampala District, the prevalence of *H. pylori* infection on the basis of presence of *H. pylori* stool antigens was 45% and ranged from 18% in Apac to 60% at Kawempe Health Centre. Previous data for adults in Uganda indicate *H. pylori* infection rates of 74% [7] and 86% [8]; however, these data were based on patients in Kampala District who had been referred for endoscopy or had cancer and benign tumors and were therefore not representative of the general population. *H. pylori* infection based on a stool antigen test was 44.3% in children twelve years and below [9] and 63% based on a serological test in children aged 1–10 years in the same low-income urban setting in Kampala City [10]. Because prevalence data from pregnant women have been found to typically reflect *H. pylori* prevalence among the general population [28–30], we believe our data provide useful information regarding the distribution of *H. pylori* infection in the country.

While the overall prevalence of *H. pylori* infection found in this study is consistent with most other studies in Africa, the variation by geographical location was unexpected. The prevalence of infection among women enrolled in Apac was significantly lower than the rates in the other locations in Uganda and was lower than rates typically reported for Africa. In a study among pregnant women recruited at ANC clinics in Pemba, Zanzibar, the prevalence of *H. pylori* infection by the ^13^C urea breath test was 17.5% and was associated with enrollment at clinics located along a major road [31]. The authors suggested that the distribution of infection reflected higher contact with the outside world experienced along the major highway. Interestingly, a recent study in Cameroun found that the prevalence of *H. pylori* infection among the Baka Pygmies who live in the rainforests of Cameroun, Congo and Gabon was much lower than among their neighbouring non-Baka [32]. The authors suggest that the Baka acquired their *H. pylori* infections from non-Baka neighbors and the frequency of *H. pylori* infection of the Baka is probably limited by population size and other demographic factors [32]. The existence of geographically localized population groups with low *H. pylori* prevalence in developing country settings that are otherwise expected to have a high prevalence was first reported in the northeastern Peninsula Malaysia where ethnic Malays, who comprise 90% of the population have an extraordinarily low *H. pylori* prevalence as compared with the Chinese or Indian populations living in the same region (reviewed by Lee et al. [33]). Similar to the Baka of Cameroun, *H. pylori* infection in Malays is related to transmission from non-Malay immigrants and the transmission of the infection has been limited by minimal contact with the immigrants as well as other factors yet to be described [33]. Apac District is in the part of northern Uganda that was, until recently, affected by civil strife. Contact with the rest of the country was limited; this is reflected in the fact that only one of the 88 women enrolled at the four health facilities located in diverse areas of Apac was not of the Lango ethnic group, while all the Langi in the study were, without exception, enrolled in Apac. Furthermore, at 65 persons per square kilometer, Northern Uganda has the lowest population density in Uganda [21]. Moreover, Apac was not markedly different from the other locations with regard to background characteristics apart from having the highest proportion of women in the lowest wealth quintile. These findings suggest that the lower population density and relative socio-economic isolation of the area are factors to consider in the low prevalence of *H. pylori* infection in this area of Uganda. Information regarding *H. pylori* infection in areas bordering Northern Uganda is scarce; a study in Sudan using the rapid urease test and culture to diagnose *H. pylori* infection found a prevalence of 16% in normal control subjects compared to 80% in patients with gastritis and 56% in patients with duodenal ulcer [34]. It would be of interest to study the epidemiology of *H. pylori* infection in the region as a whole as well as examining whether the low prevalence of H. pylori among the Langi is due to the “racial cohort” phenomenon as has been demonstrated for the Malays [33].

The positive association between *H. pylori* infection and wealth quintile obtained from univariate analysis was eliminated after adjusting for location. Moreover, Apac, which had the highest proportion of women in the lowest wealth quintile, had the lowest prevalence of *H. pylori* infection. The relationship between *H. pylori* infection and socio-economic status in developing countries is not consistent: several studies have found an inverse relationship [35–39] while Farag et al. [31] found a positive relationship. Other studies have not found any relationship [9, 40, 41]. The different contexts of low socio-economic status in these studies may account for the inconsistency: in some studies such as ours, low socio-economic status is associated with rural areas. To further illustrate, in Uganda, 2.9% and 72.7% of urban households respectively are in the lowest and highest wealth quintiles, compared to 22.5% and 12.3% of rural households respectively [22]. Farag et al. [31] also reported that women attending antenatal care clinics located at a distance from a major road had lower socio-economic status and lower rates of infection compared to women enrolled at antenatal clinics located at the main road. In other studies the context of lower socio-economic status is urban crowded living conditions [35–37, 39]. We also found a higher prevalence of *H. pylori* among the women enrolled at Kawempe Health Centre, which serves a densely-populated low-income area.

Having no formal education was positively associated with *H. pylori* infection. This finding is in agreement with other studies [37–39, 41, 42]. Education may mediate the observed association by influencing personal and household hygiene practices. Using water from rivers, lakes and streams was associated with 5-fold higher odds of infection compared to using private taps after adjusting for several background variables including location and rural/urban residence. Studies in Peru [43], Kazakhstan [35] and Brazil [37, 44] found an association between *H. pylori* infection and surface water sources, such as rivers, for household use. Fujimura et al. [45] demonstrated that river water in the natural environment could be a risk factor for *H. pylori* transmission. *H. pylori* has previously been detected in drinking water [46, 47]. The presence of *H. pylori* in water supplies might be the result of contamination from human sewage [48–51]. We postulate that the use of contaminated surface water sources with inadequate treatment at the household level is involved in the transmission of *H. pylori* infection in Uganda. Interestingly, Apac which had the highest proportion of women using water from rivers, lakes and streams had the lowest prevalence of infection, providing further support for the hypothesis of limited exposure due to “isolation” in that although these are surface water sources, they are not yet contaminated due to the relative isolation of the location.

A limitation of our study is that we used the stool antigen test, which has not been validated for the Ugandan population, to detect *H. pylori* infection. However, the stool antigen test, and specifically the Premier Platinum HpSA test has been evaluated in diverse populations [19, 52–56] and has been used widely in Africa [9, 55, 57–60]. Furthermore, the associations between *H. pylori* infection and the use of surface water sources, low educational attainment and overcrowding, have been reported in other studies thus validating our findings. Other potential limitations include not specifically excluding women who had used proton-pump inhibitors in the two weeks preceding data collection or women with hyperemesis gravidarum.

## Conclusions

In conclusion, this study found that prevalence of *H. pylori* infection in Uganda is not homogeneous but varies by geographic location and by ethnic group. This calls for population-based studies in the region to further describe the epidemiology of *H. pylori* and provides an opportunity to study the transmission dynamics of *H. pylori* infection. The use of rivers, lakes and streams in households is a risk factor for *H. pylori* infection, suggesting waterborne transmission of the infection thus highlighting the need for concerted efforts in environmental health in communities and at the household level.

## Abbreviations

ANC: Antenatal care
CI: Confidence Interval
HC: Health Centre
HpSA: H. pylori stool antigen test
OR: Odds ratio.
